# Oseltamivir-Resistant Pandemic (H1N1) 2009 Virus Infections, United States, 2010–11

**DOI:** 10.3201/eid1802.111466

**Published:** 2012-02

**Authors:** Aaron D. Storms, Larisa V. Gubareva, Su Su, John T. Wheeling, Margaret Okomo-Adhiambo, Chao-Yang Pan, Erik Reisdorf, Kirsten St. George, Robert Myers, Jason T. Wotton, Sara Robinson, Brandon Leader, Martha Thompson, Marjorie Shannon, Alexander Klimov, Alicia M. Fry, for the US Antiviral Resistance Surveillance Working Group

**Affiliations:** Centers for Disease Control and Prevention, Atlanta, Georgia, USA (A.D. Storms, L.V. Gubareva, S. Su, J.T. Wheeling, M. Okomo-Adhiambo, A. Klimov, A.M. Fry);; California Department of Public Health, Sacramento, California, USA (C.-Y. Pan);; Wisconsin State Laboratory of Hygiene, Madison, Wisconsin, USA (E. Reisdorf);; New York State Department of Health, Albany, New York, USA (K. St. George);; Maryland Department of Health and Mental Hygiene, Baltimore, Maryland, USA (R. Myers);; Minnesota Department of Health, Saint Paul, Minnesota, USA (J.T. Wotton);; Maine Department of Health and Human Services, Augusta, Maine, USA (S. Robinson);; Washington State Department of Health, Shoreline, Washington, USA (B. Leader);; Texas Department of State Health Services, Austin, Texas, USA (M. Thompson);; Delaware Health and Social Services, New Castle, Delaware, USA (M. Shannon)

**Keywords:** influenza, influenza A, H1N1 virus, pandemic (H1N1) 2009, pandemic, antiviral drug resistance, oseltamivir, oseltamivir resistance, virus, United States

## Abstract

During October 2010–July 2011, 1.0% of pandemic (H1N1) 2009 viruses in the United States were oseltamivir resistant, compared with 0.5% during the 2009–10 influenza season. Of resistant viruses from 2010–11 and 2009–10, 26% and 89%, respectively, were from persons exposed to oseltamivir before specimen collection. Findings suggest limited community transmission of oseltamivir-resistant virus.

During the 2009–2010 influenza pandemic, when pandemic (H1N1) 2009 virus was the predominant circulating influenza virus (*1*), the prevalence of oseltamivir-resistant pandemic (H1N1) 2009 viruses in the United States was 0.5%. Of the patients with oseltamivir-resistant virus infection, 89% had been exposed to oseltamivir before specimen collection (*2*). We describe patients with oseltamivir-resistant pandemic (H1N1) 2009 virus infections during the 2010–11 influenza season.

## The Study

During October 1, 2010–July 31, 2011, the Centers for Disease Control and Prevention (CDC; Atlanta, Georgia, USA) asked all state public health laboratories to submit pandemic (H1N1) 2009 virus–positive respiratory specimens and virus isolates for antiviral susceptibility testing. Laboratories were asked to provide the first 5 specimens of any type/subtype collected during each 2-week period for virus isolation. Comprehensive antiviral testing, including neuraminidase inhibition (NI) assay, was performed on all isolates, and sequencing was performed on all isolates with elevated 50% inhibitory concentration values. CDC also requested that laboratories provide 5 additional specimens every 2 weeks for pyrosequencing to identify the H275Y substitution in the neuraminidase, a change associated with oseltamivir resistance (*3*). In addition to (or instead of) participating in the national surveillance, state laboratories in California, Maine, Maryland, Minnesota, New York, Texas, and Washington performed pyrosequencing on state surveillance specimens to detect the H275Y substitution. We included those state-specific data in the national surveillance data for this report. State health departments used a standard case form to collect demographic and clinical information for patients with oseltamivir-resistant pandemic (H1N1) 2009 virus infection and their ill close contacts.

Oseltamivir resistance was determined by use of an NI assay or pyrosequencing for the H275Y substitution. For NI testing on isolates, we used the NA-Fluor kit (Applied Biosystems, Foster City, CA, USA) as described (*4*). We performed pyrosequencing, as described (*5*), on all oseltamivir-resistant pandemic (H1N1) 2009 isolates identified by NI assay to confirm the presence of the H275Y substitution. We performed pyrosequencing for H275Y, without the NI assay, to screen pandemic (H1N1) 2009 clinical specimens (*5*). For the national surveillance, NI testing was performed at CDC and pyrosequencing was performed at CDC and at state laboratories in Wisconsin, New York, and California. State laboratories followed pyrosequencing protocols provided by CDC; when possible, CDC confirmed results for resistant viruses by use of pyrosequencing and NI assay. Most oseltamivir-resistant viruses in this report were included in the weekly FluView report prepared by CDC (*6*).

We tested a total of 3,652 pandemic (H1N1) 2009 virus isolates and specimens from every state and the District of Columbia; 35 (1.0%) isolates/specimens from a total of 18 states were oseltamivir-resistant (Figure). Overall, 8 (1.3%) of 609 isolates tested by NI assay and 27 (1.0%) of 3,043 specimens tested by pyrosequencing were resistant to oseltamivir. The state-specific prevalence of oseltamivir-resistant pandemic (H1N1) 2009 viruses varied; however, the number of viruses and specimens tested also varied markedly between states, and several states submitted only a few specimens. Forty-four states submitted >20 specimens for antiviral resistance surveillance. The prevalence of oseltamivir resistance among these specimens ranged from 0% to 5.6%. None of the 609 pandemic (H1N1) 2009 isolates tested by NI assay were resistant to zanamivir. The ranges of 50% inhibitory concentration values for oseltamivir-resistant and -susceptible isolates were 166.17–230.37 nmol/L and 0.10–0.80 nmol/L, respectively.

**Figure:**
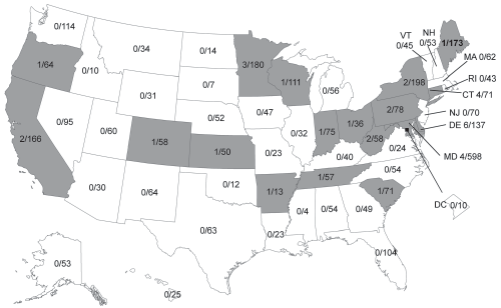
Geographic distribution of oseltamivir-resistant pandemic (H1N1) 2009 viruses in the United States, October 1, 2010–July 31, 2011. Numerators are number of oseltamivir-resistant viruses identified by state public health laboratories; denominators are number of pandemic (H1N1) 2009–positive specimens submitted by each state for susceptibility testing. Gray shading indicates states that had >1 infection with oseltamivir-resistant virus.

The median age of the 35 patients with oseltamivir-resistant pandemic (H1N1) 2009 virus infections was 33 years. Of 34 patients with available information, 26% reported receiving oseltamivir before providing a specimen for antiviral susceptibility testing (Table 1). Among 33 patients with a completed case form, 67% had at least 1 preexisting chronic medical condition, 24% had an immunocompromising medical condition, 42% required hospitalization, and 9% died. Most patients with oseltamivir-resistant virus infection for whom housing information was available lived in a single-family household. Two siblings from 1 household had oseltamivir-resistant virus infection; neither child had received oseltamivir.

**Table 1:** Characteristics of patients with oseltamivir-resistant pandemic (H1N1) 2009 virus infection, United States, October 1, 2010–July 31, 2011*

Characteristic	No. with characteristic/total no. (%), n = 35
Male sex	19/35 (54)
Exposed to oseltamivir before specimen collection†	9/34 (26)
Exposed to another person using oseltamivir	0/15 (0)
Received 2010–11 influenza vaccine	6/22 (27)
Any chronic medical conditions	22/33 (67)
Chronic pulmonary disease, including asthma	10/33 (30)
Chronic cardiac disease	6/33 (18)
Diabetes mellitus	8/33(24)
Immunocompromising condition‡	8/33(24)
Pregnancy	1/33 (3)
Other§	10/33 (30)
Lived in a single-family household	14/22 (64)
Lived in a residential facility	1/22 (4)
Others in the household/residence were ill before patient’s illness	2/15 (13)
Traveled within 7 d before illness	2/18 (11)
Hospitalized during influenza illness	14/33 (42)
ICU admission	8/14 (57)
Died	3/34 (9)

*The median age of patients was 33 y (range 1 mo–78 y). ICU, intensive care unit. †Nine patients began oseltamivir treatment prior to specimen collection; none received oseltamivir chemoprophylaxis. ‡Includes HIV/AIDS, malignancy, autoimmune disorder, solid organ transplant, stem cell transplant, and history of taking immunosuppressive therapy in the past year. §Includes morbid obesity, obstructive sleep apnea, chronic liver disease, and neurologic or developmental disorders.

All oseltamivir-resistant pandemic (H1N1) 2009 viruses were identified from specimens collected during December 2010–April 2011; the prevalence of resistance did not change significantly over time (test for trend, p = 0.18) (Table 2). In addition, the proportion of patients with oseltamivir-resistant virus infections who did not have exposure to oseltamivir before specimen collection did not change significantly over time (test for trend, p = 0.48); however, the number of specimens tested each month was small.

**Table 2:** Temporal trends of oseltamivir-resistant pandemic (H1N1) 2009 viruses and history of patient exposure to oseltamivir, by month, United States, October 1, 2010–July 31, 2011

Year and month of specimen collection	No. specimens tested	No. (%) oseltamivir-resistant specimens	No. (%) patients with oseltamivir-resistant virus but no oseltamivir exposure
2010 Oct	9	0	0
2010 Nov	17	0	0
2010 Dec	172	1 (0.6)	0
2011 Jan	1,074	6 (0.6)	4 (66.7)
2011 Feb	1,475	18 (1.2)	15 (83.3)
2011 Mar	801	9 (1.1)	6 (66.7)
2011 Apr	95	1 (1.0)	1 (100.0)
2011 May	8	0	0
2011 Jun	0	0	0
2011 Jul	1	0	0
Total	3,652	35 (1.0)	25 (73.5)*

*Oseltamivir exposure known for 34 of 35 patients.

The number of oseltamivir-resistant pandemic (H1N1) 2009 virus–infected patients was small during the 2009–10 (April 2009–September 2010) and 2010–11 influenza seasons. However, the prevalence of oseltamivir-resistant virus–infected patients was slightly higher during 2010–11 compared with 2009–10 (35/3,652 [1.0%] vs. 37/6,740 [0.5%], respectively, p = 0.02 by χ^2^ test) (*2*). Also, during 2010–11, compared with 2009–10, more patients with oseltamivir-resistant virus infection had no history of oseltamivir exposure before specimen collection (25/34 [73.5%] vs. 4/35 [11.4%], respectively; p<0.0001 by χ^2^ test).

## Conclusions

During the 2010–11 US influenza season, the prevalence of oseltamivir-resistant pandemic (H1N1) 2009 viruses remained low, and most persons with oseltamivir-resistant virus infection had no prior oseltamivir exposure. This is a notable difference from surveillance findings both globally and in the United States during the 2009–10 season, when most patients with oseltamivir-resistant pandemic (H1N1) 2009 virus infection had a history of oseltamivir exposure, and many were severely immunocompromised, a condition that may increase the risk for resistance developing during oseltamivir therapy (*2**,**7*). These data suggest a low level of community transmission of oseltamivir-resistant pandemic (H1N1) 2009 virus in the United States during the 2010–11 influenza season. The United Kingdom also reported that the proportion of oseltamivir-resistant pandemic (H1N1) 2009 virus–infected patients without prior oseltamivir exposure was higher during 2010–11 than 2009–10 (*8*).

The increase during the 2010-11 influenza season in the proportion of patients with oseltamivir-resistant pandemic (H1N1) 2009 virus infections without prior oseltamivir exposure causes concern in light of the recent history of oseltamivir resistance among seasonal influenza A (H1N1) viruses that circulated before pandemic (H1N1) 2009 virus emerged. In the United States before the 2007–08 influenza season, the prevalence of oseltamivir resistance among seasonal influenza A (H1N1) viruses was <1% (*9**,**10*). However, during the 2007–08 season, the prevalence of oseltamivir resistance among seasonal influenza A (H1N1) viruses increased to 12%, and by the 2008–09 season, resistance dramatically increased to >99% (*9**,**11**,**12*). No association was found between this increase in oseltamivir resistance and prior oseltamivir use (*11**,**13*). Oseltamivir resistance in pandemic (H1N1) 2009 and seasonal influenza A (H1N1) viruses was conferred by the H275Y substitution in the neuraminidase. However, unlike seasonal influenza A (H1N1) viruses, which retained susceptibility to the M2-blocking adamantanes (amantadine and rimantadine), >99% of circulating pandemic (H1N1) 2009 viruses are inherently resistant to adamantanes (*14*). Thus, inhaled zanamivir or investigational drugs, such as intravenous zanamivir, are the only treatment options for patients with oseltamivir-resistant pandemic (H1N1) 2009 virus infection.

Our conclusions are limited by the small number of patients with oseltamivir-resistant pandemic (H1N1) 2009 virus infection. Variability in state surveillance and the number of specimens tested from each state may also have limited the representativeness of our data. Despite these shortcomings, our findings emphasize the importance of ongoing surveillance for oseltamivir-resistant pandemic (H1N1) 2009 viruses in the United States and globally and of close monitoring for changes in the epidemiology of oseltamivir resistance among pandemic (H1N1) 2009 viruses. Updated information about antiviral resistance in influenza viruses in the United States is available at www.cdc.gov/flu/professionals/.
